# Margin reduction and optimal prescription isodose model for liver stereotactic radiotherapy with respiratory motion

**DOI:** 10.1002/acm2.70455

**Published:** 2026-01-07

**Authors:** Daisuke Kawahara, Hirokazu Masuda, Takuya Wada, Misato Kishi, Tsuyoshi Katsuta, Yuji Murakami

**Affiliations:** ^1^ Department of Radiation Oncology, Institute of Biomedical & Health Sciences Hiroshima University Hiroshima Hiroshima Japan; ^2^ Radiation Therapy Section, Department of Clinical Support Hiroshima University Hospital Hiroshima Hiroshima Japan

**Keywords:** 4DCT, internal margin, stereotactic body radiotherapy

## Abstract

**Purpose:**

This study aimed to establish a framework for optimizing planning target volume (PTV) margins in liver SBRT using virtual 4DCT (v4DCT), by introducing the concept of the Dosimetric Coverage Amplitude (DCA) to quantify motion tolerance and deriving an Optimal Margin (OM) that balances tumor coverage with normal‐tissue sparing.

**Methods:**

VMAT plans were developed using a whole‐body phantom with a virtual tumor, ensuring that the prescription dose corresponded to D_95%_ of the PTV. The 60%–80% isodose levels were defined relative to the maximum dose to represent alternative prescription surfaces. v4DCT simulated free‐breathing conditions across 10 respiratory phases to generate virtual four‐dimensional radiotherapy (v4DRT) dose distributions. The DCA was defined as the maximum respiratory amplitude at which GTV dose coverage (D100% or D99%) was maintained. Based on DCA analysis, the OM was determined as the clinically applicable margin derived from the isodose plan that satisfied the DCA condition and minimized normal tissue dose.

**Results:**

DCA analysis revealed that GTV dose coverage was maintained for respiratory motion amplitudes up to 1.9–2.2 times larger than the conventional PTV margin of 10 mm. Taking into account GTV dose coverage, average GTV dose relative to the 80%‐static reference, and normal‐tissue dose, the 60% isodose plan was identified as the optimal prescription level. From this plan, the OM was calculated as 5.6 mm, representing a 44% reduction compared with the conventional 10‐mm PTV, while maintaining GTV coverage and minimizing liver dose. This margin reduction was attributed to the steeper dose gradient associated with prescribing to a lower isodose level. The v4DCT approach enabled the generation of multiple respiratory phase images, allowing quantitative evaluation of motion‐induced dose distribution and optimization of margins based on respiratory variability.

**Conclusion:**

The proposed v4DCT‐based framework demonstrated that the 60% isodose plan provided the optimal balance between tumor coverage and normal‐tissue sparing, yielding an OM of 5.6 mm (44% reduction compared with the conventional 10‐mm PTV). This approach offers a clinically applicable strategy for margin reduction in liver SBRT while maintaining robust dosimetric coverage.

## 1 INTRODUCTION

Stereotactic body radiation therapy (SBRT) is a promising local treatment for liver cancer that demonstrates remarkable efficacy in achieving local control of both primary liver tumors and metastases of other origins.^1^, ^2^ Improvement in local control is associated with a smaller tumor volume and a higher target dose.^2^, ^3^ Thus, in recent clinical studies, volume prescription using the volumetric modulated arc therapy (VMAT) technique, which delivers a tumor dose as high as possible while maintaining liver constraints, has been used.^3^, ^4^, ^5^, ^6^ The prescription dose covers at least 95%–99% of the planning target volume (PTV), while the gross tumor volume (GTV) typically receives a higher dose than the prescription level to ensure effective tumor control.

To deliver accurate doses, it is necessary to accurately capture tumor locations and apply an optimal PTV margin for respiratory motion. For liver SBRT, the management of respiratory tumor motion, such as breath‐holding, dynamic tracking, abdominal compression, and respiratory synchronization, is available. Breath‐hold techniques and gated radiotherapy, although precise, require patient cooperation and advanced equipment, potentially limiting their use in certain populations. Respiratory tracking offers a sophisticated solution for matching real‐time tumor motion, although its complexity and cost may be prohibitive. Although free breathing may decrease the overall treatment duration, these methods result in a higher dose to the surrounding healthy tissue owing to the increased PTV.^7^

According to the International Commission on Radiation Units and Measurements (ICRU), the optimal PTV margin is defined as the net effect of all possible geometrical variations and inaccuracies to ensure that the prescribed dose is absorbed in the clinical target volume (CTV).^8^, ^9^ The PTV margin is generally defined to be considered with the internal margin volume (ITV) margin and setup margin. The ITV is defined as the target volume for the entire range of tumor motion across the respiratory cycle.

In our previous work, this parameter was termed the dosimetric internal margin (DIM).^10^ In this study, to avoid confusion with clinical geometric margins, we redefine it as the Dosimetric Coverage Amplitude (DCA), which represents a maximum motion amplitude rather than a margin. In contrast to these classical geometric definitions, the present study does not construct a conventional ITV; instead, respiratory motion is represented through the DCA, from which the optimal margin (OM) is derived. The OM is then derived from the DCA‐optimal plan. However, DCA alone does not optimize normal tissue sparing. To address this, we introduce the OM, defined by selecting an appropriate isodose level (e.g., 60%–80%) that balances GTV coverage with minimal OAR irradiation, thus refining margin selection in a clinically feasible way.^11^

Katsuta et al. proposed a novel simulation designed to evaluate the dosimetric effect of diaphragmatic motion on four‐dimensional volumetric modulated arc therapy (4D VMAT) for the treatment of oesophageal cancer.^12^ The simulation integrates advanced imaging and planning techniques to accurately model the diaphragm movement during the respiratory cycle, thus providing a more realistic assessment of the radiation dose distribution by creating a virtual four‐dimensional computed tomography (v4DCT) in both the target tumor and surrounding OARs. Although Katsuta et al. focused on esophageal tumors with a maximum amplitude of 12 mm, their methodology was adapted for hepatic tumors, which can exhibit larger respiratory amplitudes of up to 24 mm. Previous studies have reported that respiratory‐induced motion of liver tumors can reach up to 24 mm in the superior–inferior direction.^13^ The differences in motion amplitude necessitate careful adaptation, as hepatic tumors often experience greater displacement due to their anatomical location and proximity to the diaphragm. This study evaluates the validity of this approach for larger motion amplitudes, ensuring its applicability to liver SBRT. Margin reduction in liver SBRT is critical to adhere to hepatic dose constraints and minimize exposure to OARs such as the stomach, intestines, and uninvolved liver. Reducing the PTV margin allows for dose escalation to the tumor while sparing healthy tissue, ensuring the treatment's efficacy and safety. This study proposes an improved 4D VMAT system to evaluate 4‐dimensional dose calculation with v4DCT for liver SBRT patients to evaluate the dose in the target and OARs. In addition, the interplay effect—caused by the interaction between tumor motion and dynamic radiation delivery—can lead to dose heterogeneity, particularly in VMAT‐based SBRT.^14^ Previous studies indicate that its impact depends on motion amplitude and delivery technique, making it essential to assess its influence on liver SBRT.^15^, ^16^

This study explicitly extends Katsuta et al.’s model to liver tumors by incorporating larger respiratory amplitudes and adjusting the approach to consider liver‐specific anatomical and dosimetric constraints. Optimizing the prescription isodose volume plays a key role in achieving precise dose delivery to the target while minimizing exposure to OARs. This is crucial in liver SBRT, where dose escalation must be balanced against the potential for hepatic toxicity. Moreover, this study also examines the interplay effect through phantom‐based verification, ensuring that the optimized margin strategy maintains robust tumor dose coverage under respiratory motion. By fine‐tuning the isodose prescription, we ensure that the mean dose remains equivalent to the stationary dose, allowing for effective margin reduction while maintaining treatment accuracy.

## 2 MATERIALS AND METHODS

Figure S1 illustrates the conceptual workflow for constructing and evaluating the virtual four‐dimensional radiotherapy (v4DRT) framework based on a virtual 4DCT (v4DCT) for liver SBRT. This workflow consists of four main components, corresponding to Sections 2A–2D of the Methods:

Section 2A: Construction of baseline treatment plans using conventional ITV = PTV margins, applied to multiple prescription isodose levels (60%, 70%, and 80%).

Section 2B: Generation of patient‐specific v4DCTs with a range of simulated respiratory amplitudes. These v4DCTs were used to deform the dose distributions and accumulate the resulting v4DRT dose distributions for each amplitude.

Section 2C: Evaluation of the accumulated dose distributions to determine the following:

Dosimetric coverage amplitude (DCA): Defined as the maximum respiratory amplitude at which the accumulated dose still satisfies coverage criteria for the GTV (*D*_100%_ or *D*_99%_ ≥ prescribed dose). DCA quantifies motion tolerance under each isodose plan.

Optimal isodose plan: Among the tested plans (60%, 70%, and 80%), the optimal isodose plan is the one that maintains sufficient GTV coverage up to the DCA while minimizing the dose to surrounding normal tissue. This plan represents a clinically practical trade‐off between tumor coverage and OAR sparing.

Optimal margin (OM): Defined as the spatial extent (irradiated volume) corresponding to the optimal isodose boundary. Unlike geometric expansions used in conventional ITV+PTV strategies, the OM is derived from the optimal plan identified through DCA analysis and reflects the minimal necessary margin informed by motion robustness and dose distribution. The OM can thus be interpreted as a motion‐informed, dose‐based alternative to conventional margins.

Section 2D: Evaluation of the interplay effect under the selected OM and optimal isodose plan, using phase‐resolved v4DRT simulation.

In this study, the OM was evaluated based on the GTV defined on a free‐breathing average CT image. The accumulated dose was calculated using the v4DCT derived from deforming the free‐breathing dataset according to phase‐resolved motion amplitudes. The proposed OM concept is intended to replace the ITV typically generated to encompass tumor motion. Thus, the margin defined by the OM is applied directly to the CTV, eliminating the need to create an intermediate ITV. A conventional PTV margin (e.g., 3–5 mm for setup uncertainty) may still be applied beyond the OM in clinical implementation. This approach simplifies the planning process by defining the OM derived from dose‐based criteria, instead of geometric motion range.

This framework provides a structured approach for integrating respiratory motion analysis (via DCA), dose distribution adaptation (via optimal isodose selection), and margin definition (via OM), allowing comparison with conventional margin strategies in terms of both tumor control and normal tissue sparing.

### 2.1 Treatment planning

A whole‐body human‐equivalent phantom (Whole‐Body Phantom PBU‐60; KYOTOKAGAKU, Kyoto, Japan) was used. Computed tomography (CT) was performed using a 16‐detector row spiral CT scanner (LightSpeed RT16; GE Healthcare, Little Chalfont, UK). The slice thickness and interval were 2.0 mm. Treatment planning was performed using RayStation ver. 10.0.1 (RaySearch Medical Laboratories, Stockholm, Sweden).

The virtual tumor was modeled as a spherical geometry with a diameter of 1 cm. It was placed in the right lobe of the liver. The virtual tumor was defined as the GTV, and the CTV margin was 0 mm around the GTV in the left–right (LR), anterior–posterior (AP), and cranio‐caudal (CC) directions, following the NRG‐BR001 protocol.^17^ To create the initial planning volume for all plans, a fixed isotropic 10‐mm expansion was applied to the gross tumor volume (GTV). This expansion was used solely as a reference geometric planning margin and should not be interpreted as a classical ITV derived from patient‐specific respiratory motion.^18^ Instead, it served as a standardized initial PTV from which prescription‐isodose plans (60%, 70%, and 80%) were generated. Unlike conventional ITV construction—where respiratory motion is explicitly assessed and incorporated—the aim of this study was to determine how well a fixed geometric margin maintains dose coverage under simulated respiratory motion, and to derive the DCA representing the largest motion amplitude that preserves adequate GTV dose. Based on the robustness associated with different prescription isodose levels, we defined the OM as the smallest irradiated region required to satisfy the DCA condition. Thus, in this framework, the OM functions as a dose‐based, motion‐informed alternative to the classical ITV. Treatment planning used a 10‐MV flattening filter‐free beam of the TrueBeam linear accelerator (Varian Medical Systems, Palo Alto, USA), as shown in Figure S2. A dose of 40 Gy in four fractions was prescribed.^19^ VMAT plans were generated using a whole‐body phantom with a virtual tumor. For all plans, the dose distribution was normalized such that 95% of the PTV received 100% of the prescribed dose. The prescription isodose surface was then defined as a percentage of the maximum dose. Based on this definition, three different plans were created: 60%, 70%, and 80% isodose prescriptions. The 80% isodose plan corresponds to the conventional prescription level, whereas the 60% and 70% plans represent lower prescription levels associated with steeper dose gradients. The gantry and collimator angles were set to 0°–180°(counterclockwise) and 10°, respectively.

### 2.2 Virtual 4D radiotherapy with virtual 4D computed tomography

**FIGURE 1:**
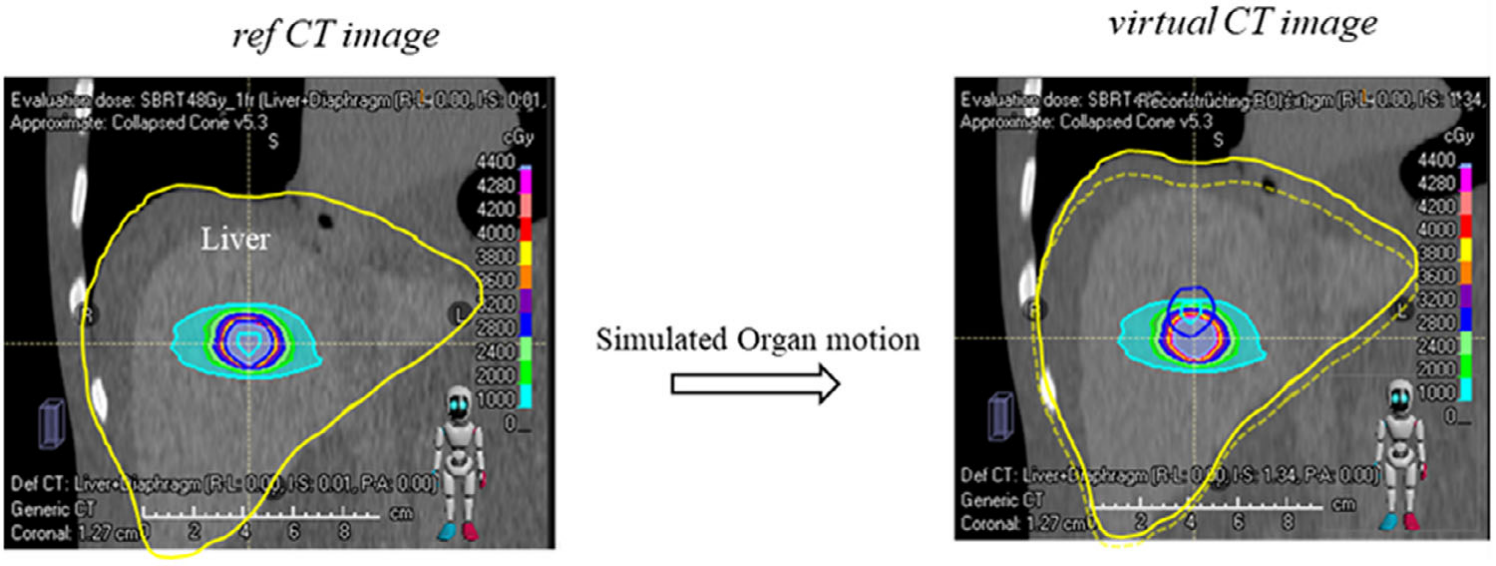
Synthesis of the virtual CT images from the reference CT images.

The static CT images were deformed into v4DCT images through the simulated‐organ motion (SOM) mode on RayStation,^20^ with the entire liver deformed in the superior direction with vertebral fixation, as shown in Figure 1. Figure S3 shows a schematic of the creation of the virtual 4DCT and virtual 4DRT, calculated using the dose distribution with the deformed virtual 4DCT.

A v4DCT image comprised 10 respiratory phases, whose amplitudes were calculated using the following equation^21^:

(1) $$z\left(t\right)=z_{0}-b\cos^{2n}\left(\pi t/\tau \right),$$

where $z_{0}$ is the exhale coordinate, τ is respiratory period, *b* is respiratory amplitude, and *n* is a fitting parameter indicating asymmetry of the breathing pattern. The current study assumed a symmetrical respiratory cycle and used *n *= 1. The value of the *z*‐axis was determined for each phase as $z_{0}$ = *b*, and the amplitude of each phase was calculated as *t* = 0.1τ, 0.2τ,…, 1.0τ, whereas the motion amplitude was defined separately as a spatial displacement in millimeters (mm). To avoid confusion, we clarified that the amplitude represents displacement and is not calculated from time. The dose distribution of the v4DCT was calculated using the reference dose. The v4DCT images were generated by assuming free breathing with amplitudes ranging from 1 to 24 mm. The calculated dose in v4DCT was deformed onto the reference CT images, and v4DRT was created by merging the deformed doses.^12^ The doses in v4DRT were calculated using the probability of existence of each respiratory phase for *n* = 1.^12^ The cumulative dose under motion was calculated by deformably accumulating phase‐specific dose distributions across the respiratory cycle using deformable image registration–based dose‐warping

### 2.3 Evaluation for Virtual 4D and optimal margin

The DCA was defined as the maximum respiratory amplitude at which the GTV maintained adequate dose coverage (*D*_100%_ or *D*_99%_) under motion (Figure S4). Mathematically, this can be expressed as:

(2) $$\mathrm{DCA}=max_{amplitude}\;\left\{z\left(t\right)|D_{GTV}\left(t\right)\geq D_{crit}\right\}.$$

where z(t) represents the tumor displacement due to respiratory motion at time 𝑡, and 𝐷crit denotes the coverage criterion (*D*_100%_ or *D*_99%_). Thus, DCA represents the tolerable range of respiratory motion in which adequate target coverage is preserved. Based on the DCA analysis, a DCA‐optimal plan was identified among the three prescription isodose levels (60%, 70%, and 80%). For clarity, we denote the DCA obtained from the 80% prescription isodose plan as DCA80%, and similarly DCA70% and DCA60% for the other plans. The DCA‐optimal plan was defined as the lowest isodose level that satisfied all of the following conditions:

1. Maintained GTV dose coverage during respiratory motion up to the amplitude defined by DCA,

2. Preserved the average GTV dose under motion ($D_{average}^{x\%,resp}$) t a level greater than or equal to the average GTV dose of the 80% plan in the static phase ($D_{average}^{80\%,resp}$), and

3. Reduced unnecessary dose to adjacent normal tissues (liver, stomach, and intestines) compared with the 80% plan.

The OM was then calculated from the DCA‐optimal plan as the clinically applicable margin. Specifically, the conventional 10‐mm PTV margin was scaled according to the ratio of the observed respiratory displacement ($\Delta_{resp}$) to the $DCA_{opt}$:

(3) $$\mathrm{OM}=\frac{PTV_{margin}}{DCA_{opt}}\times \Delta_{resp}$$

where $PTV_{margin}$ = 10 mm in this study. In summary, the DCA quantified the maximum motion amplitude tolerable for adequate tumor coverage, the DCA‐optimal plan was the prescription isodose level that fulfilled the DCA condition while minimizing OAR dose, and the OM was the final clinically applicable margin derived from this plan.

### 2.4 Assessment of the interplay effect

A water‐equivalent cubic phantom (I'mRT Phantom, IBA Dosimetry, Schwarzenbruck, Germany) was set on the QUASAR respiratory motion platform (Modus Medical Devices Inc., London, Canada) and irradiated with the treatment plan using the OM and the optimal isodose plan determined in Section B. The interplay effect was evaluated by the difference in the dose distribution during the respiratory motion at different initial respiratory phases of –π/2 and π/2 with the same initial breathing phase with the film measurement, as shown in Figure S5. The dose difference between the plan and measurement was compared by gamma analysis using DoseLab Pro software (Mobius Medical Systems, Houston, TX, USA). Gamma analysis was performed with a 3%/3 mm global criterion.

## 3 RESULTS

### 3.1 GTV dose coverage and DCA evaluation

**FIGURE 2:**
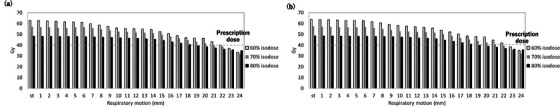
$D_{100\%}$ of the gross tumor volume (GTV) with the stationary phase and respiratory motion at the amplitude of 1–24 mm for the 60% isodose plan, 70% isodose plan, and 80% isodose plan (a). The $D_{99\%}$ of the GTV with the stationary phase and respiratory motion at the amplitude of 1–24 mm for the 60% isodose plan, 70% isodose plan, and 80% isodose plan (b).

Figure 2a shows the accumulated GTV dose indexes of $D_{100\%}$ with respiratory motion ranging from 1 to 24 mm for the 60%, 70%, and 80% isodose plans. The $D_{100\%}$ of the GTV decreased with increasing respiratory amplitude for all isodose plans. The DCA values corresponding to $D_{100\%}$ were 18, 21, and 22 mm for the 80%, 70%, and 60% plans, respectively. Figure 2b shows the accumulated GTV dose indexes of $D_{99\%}$ with respiratory motion ranging from 1 to 24 mm for the 60%, 70%, and 80% isodose plans. The DCA values corresponding to $D_{99\%}$ were 19, 21, and 22 mm for the 80%, 70%, and 60% plans, respectively. In both $D_{100\%}$ and $D_{99\%}$ evaluations, the DCA tended to be larger for lower isodose levels, reflecting the higher central dose in the tumor.

### 3.2 Optimal isodose plan and OM determination

**FIGURE 3:**
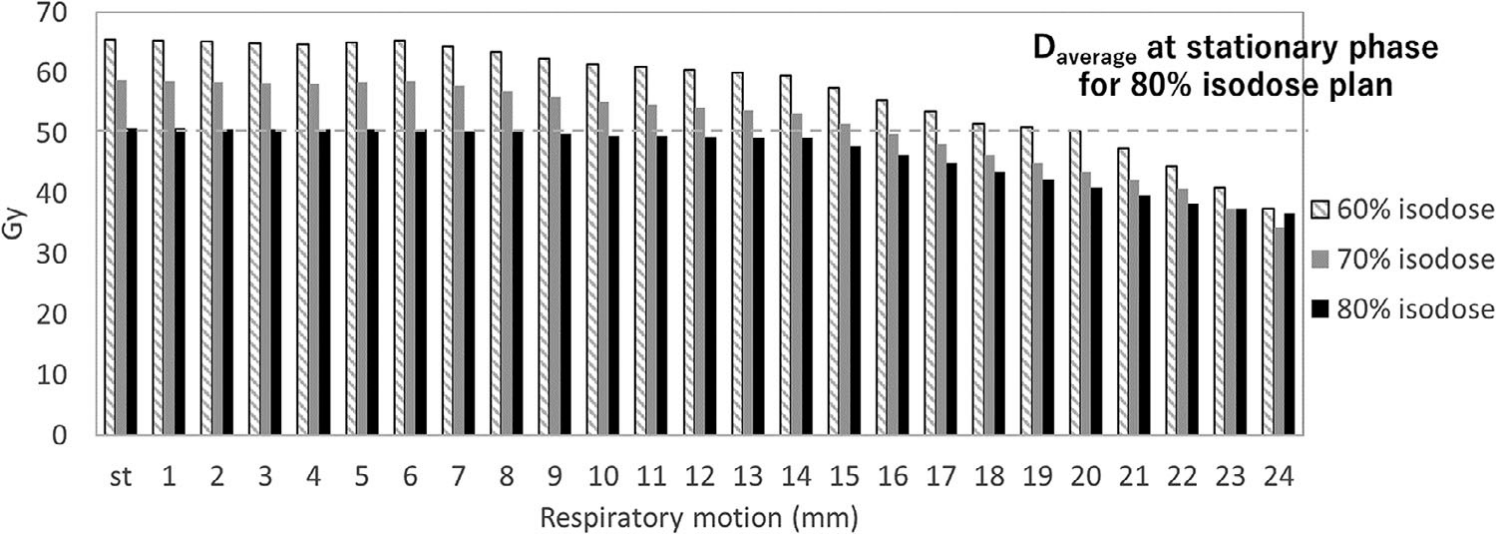
$D_{average}$ of the GTV with the stationary phase and respiratory motion at the amplitude of 1–24 mm for the 60% isodose plan, 70% isodose plan, and 80% isodose plan.

Figure 3 shows the accumulated CTV dose indices of 𝐷average with respiratory motion ranging from 1 to 24 mm for the 60%, 70%, and 80% isodose plans. The $D_{average}$ of the GTV at the stationary phase for the 80% isodose plan was 50.8 Gy, which served as the reference for comparison across the different isodose levels. With increasing respiratory amplitude, the average dose to the GTV decreased for all prescription levels, reflecting the expected blurring of dose distribution under motion. The maximum respiratory amplitudes that preserved the reference GTV dose were 15 mm for the 70% isodose plan and 19 mm for the 60% isodose plan, respectively, indicating that lower isodose prescriptions allowed tolerance of larger motion ranges. These findings were consistent with the trend observed in the *D*_100%_ and *D*_99%_ evaluations, where the DCA tended to be larger at lower isodose levels, owing to the higher central dose in the tumor.

### 3.3 Normal tissue: Peripheral dose evaluation

Table 1 shows the cumulative $D_{0.5-2.0cm}$ that is the dose received by normal tissue 0.5–2.0 cm from the PTV for both the stationary and respiratory motion phases, with amplitudes ranging from to 1 to 24 mm, across the 60%–80% isodose plans. In the comparison of the $D_{0.5-2.0cm}$ with and without respiratory motion, it was smaller with the respiratory motion for all isodose plans. In particular, the magnitude of the reduction was more pronounced for larger respiratory amplitudes. The $D_{0.5-2.0cm}$ with the respiratory motion was larger with a higher isodose plan. In the comparison of the $D_{0.5-2.0cm}$ at the stationary phase for the $D_{80\%}$ isodose plan and the respiration amplitude of the $DCA^{80\%}$ for the 70% and 60% isodose plans, the $D_{0.5-2.0cm}$ at the respiration amplitude of the $DCA^{80\%}$ for the 70% and 60% isodose plans was smaller.

**TABLE 1:** $D_{0.5-2.0cm}$ (Gy) with the stationary phase and respiratory motion at the amplitude of 1–24 mm for the (a) 60% isodose plan, (b) 70% isodose plan, and (c) 80% isodose plan.

		
		Amplitude (mm)											
(a)	Static	2	4	6	8	10	12	14	16	18	20	22	24
*D*_0.5_ _cm_	41.54	41.46	41.38	39.04	38.31	37.58	37.65	37.72	35.98	34.24	34.08	33.80	33.77
*D*_1.0_ _cm_	30.61	30.12	29.63	29.89	29.13	28.36	28.71	29.05	27.73	26.40	26.60	25.95	25.30
*D*_2.0_ _cm_	23.36	22.21	21.06	21.42	20.93	20.43	20.79	21.15	20.14	19.13	19.25	19.01	18.77

													
		Amplitude (mm)											
(b)	Static	2	4	6	8	10	12	14	16	18	20	22	24
*D*_0.5_ _cm_	38.21	37.91	37.60	37.81	36.77	35.73	35.70	35.67	33.37	31.06	31.16	31.26	30.96
*D*_1.0_ _cm_	32.19	32.08	31.96	31.16	30.19	29.21	29.17	29.12	27.37	25.62	25.09	24.55	24.16
*D*_2.0_ _cm_	25.02	24.80	24.57	24.13	23.13	22.12	21.88	21.63	20.64	19.65	18.83	18.00	17.92

													
		Amplitude (mm)											
(c)	Static	2	4	6	8	10	12	14	16	18	20	22	24
*D*_0.5_ _cm_	42.00	41.32	40.65	40.55	39.29	39.20	39.10	39.04	38.99	38.93	38.87	38.55	38.22
*D*_1.0_ _cm_	32.48	32.42	32.36	32.31	32.24	32.19	32.14	31.81	31.48	31.14	30.81	30.61	30.41
*D*_2.0_ _cm_	21.31	21.23	21.15	21.28	20.98	21.12	21.25	21.08	20.91	20.74	20.57	20.47	20.37

### 3.4 Normal tissue: Normal liver dose evaluation

Table 2 shows the cumulative V_20Gy_ and V_5Gy_ of the normal liver for both the stationary and respiratory motion phases, with amplitudes ranging from 1 to 24 mm, across the 60%–80% isodose plans. The V_5Gy_ of the normal liver increased with larger respiratory amplitudes, rising by approximately 0.7%–0.8% across all isodose plans. In contrast, the V_20Gy_ of the normal liver decreased slightly (by about 0.9%–1.8%) with larger respiratory amplitudes, but always remained within the acceptable dose constraint of 1.1%. When comparing across plans, both V_5Gy_ and V_20Gy_ were consistently lower in the 60% plan than in the 70% or 80% plans. Furthermore, comparing the stationary phase of the 80% isodose plan with the amplitudes corresponding to $DCA^{80\%}$ in the 70% and 60% plans, the V_5Gy_ was smaller for the 70% and 60% plans, while the V_20Gy_ was slightly higher, though still within 1.1%

**TABLE 2:** $V_{20Gy}$ and $V_{5Gy}$(%) of the normal liver with the stationary phase and respiratory motion at the amplitude of 1–24 mm for the (a) 60% isodose plan, (b) 70% isodose plan, and (c) 80% isodose plan.

		
		Amplitude (mm)											
(a)	Static	2	4	6	8	10	12	14	16	18	20	22	24
Liver‐GTV V_5Gy_	1.04	1.29	1.54	1.71	1.82	1.93	2.13	2.32	2.33	2.33	2.31	2.53	2.71
Liver‐GTV V_20Gy_	9.72	9.85	9.98	9.86	9.79	9.72	9.82	9.91	9.65	9.38	9.72	9.33	9.00

													
		Amplitude (mm)v											
(b)	static	2	4	6	8	10	12	14	16	18	20	22	24
Liver‐GTV V_5Gy_	1.23	1.50	1.76	1.94	2.06	2.17	2.38	2.58	2.58	2.57	2.68	2.78	3.00
Liver‐GTV V_20Gy_	10.34	10.45	10.56	10.47	10.38	10.28	10.40	10.51	10.24	9.96	9.93	9.90	9.65

													
		Amplitude (mm)											
(c)	static	2	4	6	8	10	12	14	16	18	20	22	24
Liver‐GTV V_5Gy_	1.17	1.43	1.68	1.78	1.94	2.09	2.31	2.53	2.55	2.56	2.69	2.82	2.90
Liver‐GTV V_20Gy_	10.94	10.97	10.99	10.84	10.72	10.59	10.68	10.76	10.44	10.11	10.07	10.02	10.00

Based on these results, the DCA values corresponding to the 80% isodose plan were 18 and 19 mm for the *D*_100%_ and *D*_99%_ of the GTV, respectively. When considering the average GTV dose relative to the 80%‐static reference, coverage was maintained up to 15 mm with the 70% plan and up to 19 mm with the 60% plan. In other words, the amplitudes satisfying both the DCA reference of the 80% plan and the average dose criterion were 15 mm for the 70% plan and 18 mm for the 60% plan. Comparing OAR doses at these amplitudes, the peripheral normal‐tissue dose (*D*_0.5_ _cm_– *D*_2.0 cm_) and normal liver dose were consistently lower with the 60% plan. Taken together, these findings demonstrate that while both 70% and 60% plans met the tumor coverage requirements, the 60% isodose plan offered the most favorable balance between motion robustness and normal tissue sparing, and was therefore identified as the DCA‐optimal plan.

### 3.5 Interplay effect

Figure S6 shows the dose profile calculated by the v4DRT and measured by the film in the sagittal plane on the I'mRT Phantom with the treatment plan using the OM of 18 mm and optimal isodose plan of 60%. The gamma passing rate was greater than 99.7% for the different initial respiratory phases. The dose profile of the v4DRT showed good agreement with the measurements.

## 4 DISCUSSION

Although classical ITVs are typically constructed by encompassing the geometric range of respiratory motion, the present study did not generate a conventional ITV based on 4DCT phase contours. Instead, respiratory motion was evaluated through the DCA, and the OM was derived as a dose‐based, motion‐informed alternative to the classical ITV. This distinction is important for interpreting the results below, as the analysis focuses on the dosimetric robustness of different prescription isodose levels rather than on geometric ITV construction.

In the clinic, the ITV is often generated using the amplitude of the tumor movement during respiration or the contouring of GTVs in up to 10 phases of a 4DCT scan.^22^ However, dose distribution is not considered when creating an ITV. Masuda et al. proposed a measurement‐based dosimetric optimal margin with an 80% isodose plan. Although margin reduction was possible, the dose to the OAR and the average dose to the tumor were not investigated.^10^ They reported a limitation in that the DCA was analyzed using a simple homogeneous phantom. We developed a novel framework to evaluate the dosimetric impact, including the dose to the tumor and OARs, and DCA in patient CT images according to the respiratory motion for different isodose plans.

The $DCA^{80\%}$ was 18 mm for *D*_100%_ of the GTV and 19 mm for *D*_99%_ of the GTV, which were encompassed within the tolerance ranges of the 70% and 60% plans. When considering the average GTV dose relative to the 80%‐static reference, coverage was maintained up to 15 mm with the 70% plan and up to 19 mm with the 60% plan. Therefore, the amplitudes satisfying both the DCA reference of the 80% plan and the average dose criterion were 15 mm for the 70% plan and 18 mm for the 60% plan. Moreover, importantly, at these amplitudes, peripheral normal‐tissue doses were consistently lower with the 60% plan than with higher isodose prescriptions. Accordingly, the 60% plan was identified as the DCA‐optimal plan. Applying Equation (3), the corresponding OM was 5.6 mm, representing a 44% reduction compared with the conventional 10‐mm PTV. Our findings offer significant advancements over conventional ITV delineation methods, which have predominantly focused on simple measurements of tumor motion. The proposed framework demonstrates the feasibility of reducing tumor motion margins while maintaining the average dose to the tumor. In SBRT, tumor control may depend not only on the prescribed dose but also on intratumoral hotspots, which could enhance biological effectiveness. Owen et al. emphasized the critical role of elevated doses within the GTV in achieving favorable outcomes.^23^ Although we did not directly compute the BED, the reduced OM based on lower prescription isodose levels (e.g., 60%) inherently increases the central dose due to steeper dose gradients. This adaptation significantly affects dose distribution, as evidenced by the consistently lower V_5Gy_ of normal liver in the 60% plan compared with the 70% and 80% plans (*p* < 0.001 for all pairwise comparisons). In contrast, no significant difference was observed in V_20Gy_ of normal liver (*p* = 0.60 for 60% vs. 80%, *p* = 0.19 for 70% vs. 80%, and *p* = 0.06 for 60% vs. 70%), indicating that high‐dose exposure remained stable within the acceptable constraint threshold of 1.1%. For liver SBRT, minimizing the dose to OARs is crucial, protecting the uninvolved liver tissue from central lesions and the adjacent gastrointestinal OARs from peripheral tumors. Schefter et al. introduced the ‘critical volume model’ from a phase I trial, highlighting the importance of sparing a significant volume of normal liver from high radiation doses.^24^ Garnet et al. investigated the tumor control probability by comparing the dose distribution using a PTV with and without consideration of respiratory motion.^25^ If respiratory control allows for reduction of the PTV margin, radiation doses can be focused more on the tumor, thereby minimizing the exposure of healthy tissues. This study is aligned with the evolving landscape of radiation therapy in which dynamic imaging and patient‐specific motion are integral to the planning process. By addressing the dose‐dependent effects on OARs adjacent to tumors experiencing respiratory motion, our study contributes to the broader goal of enhancing radiation therapy efficacy and patient safety.

In considering how this framework may be translated into clinical practice, it is important to clarify the potential role of v4DCT in this context. We envision two complementary functions. First, v4DCT provides a controlled and quantitative platform for generating motion‐simulated images with predefined respiratory amplitudes, allowing the systematic evaluation of motion tolerances and the derivation of amplitude‐dependent optimal margins. In this sense, v4DCT serves as a model‐building tool that supports the development of standardized margin recommendations—such as amplitude‐to‐OM relationships—based on reproducible simulations rather than patient‐by‐patient trial planning. Second, v4DCT may be used as a practical planning option when a full 4DCT acquisition is not available. If patient‐specific respiratory amplitude can be estimated using cine imaging, fluoroscopy, or surface surrogates, this information can be incorporated into a v4DCT to approximate motion‐affected dose distributions and guide the selection of an appropriate OM. In this way, v4DCT has the potential to supplement or partially replace 4DCT in selected clinical scenarios. The detailed evaluation of how accurately v4DCT reproduces organ‐level motion, including normal liver deformation, is outside the scope of the present study and is addressed in the limitations that follow. Nevertheless, these two roles position v4DCT as both a foundation for generalizable motion–margin models and a potential clinical resource in settings where full 4DCT is not feasible.

This study had several limitations. First, only homogeneous regions of the liver were evaluated. Future research will include inhomogeneous cases and other anatomical sites such as lung tumors. Second, respiratory motion was modeled using a sinusoidal waveform, which may not fully represent clinical patterns such as hysteresis, irregular cycles, or prolonged expiration. Incorporating patient‐specific motion trajectories derived from cine imaging or 4DCT will be required to enhance realism. Baseline shifts also contribute to motion‐related uncertainty. Zeng et al. reported baseline drifts of 1–2 mm in LR, SI, and AP directions, which may influence margin estimation beyond the ITV‐based geometric approach. Although the present framework did not explicitly model baseline drift, future work will extend v4DCT by incorporating stochastic and systematic baseline components, supported by mid‐treatment verification imaging and adaptive motion modeling.^26^ In addition, this study evaluated normal liver dose within 0.5–2 cm beyond the planning boundary, corresponding to the region traversed by respiratory motion up to approximately 20 mm. However, detailed analyses of cases where organs at risk (OARs) directly about the tumor were not performed. Incorporating OAR proximity into patient‐specific OM estimation represents an important area for future work. The proposed OM also differs from classical geometric margin recipes such as van Herk's formulation, which models setup uncertainty and penumbra broadening. Because the present study isolated the dosimetric effect of respiratory motion under a fixed setup margin, the OM should be interpreted as a motion‐specific dosimetric margin rather than a comprehensive PTV margin. Integration of setup uncertainty and anisotropic LR/AP/CC motion into the OM framework is planned for future studies. Furthermore, this study did not compare the OM with the mid‐ventilation approach, which derives geometric margins from time‐averaged tumor motion. Combining OM‐based dose robustness with mid‐ventilation–based geometric modeling may enhance individualized margin design. Finally, although a fixed 10‐mm initial expansion (ITV = PTV) was used for plan construction, the relative trends in OM are expected to remain stable across different initial margins because OM is derived from the relative motion–dose relationship. Future work should explore sensitivity to alternative initial geometries and incorporate probability‐based motion distributions to generalize margin estimation across diverse clinical scenarios.

In summary, our study represents a significant advancement in the field of radiation therapy by providing a holistic and flexible approach for ITV determination. Through meticulous dosimetric analysis and consideration of respiratory motion, we offer a methodology that enhances tumor‐targeting precision, minimizes radiation exposure to OARs. In clinical practice, the OM can replace the ITV component of the PTV, while a setup margin may still be added depending on institutional protocols. Our work not only builds upon, but also significantly refines previous methodologies, paving the way for more personalized and effective radiation therapy protocols.

## 5 CONCLUSION

We found that the planning target volume margin required to compensate for respiratory tumor motion could be reduced by 44% compared with the conventional 10‐mm PTV, by adopting the 60% isodose prescription, while simultaneously reducing the dose to organs at risk adjacent to the target. Importantly, the average target dose achieved with the 60% plan under respiratory motion remained comparable to that of the 80% plan in the stationary phase, confirming the robustness of this approach. The proposed framework therefore provides a clinically applicable method to derive the Optimal Margin (OM) from v4DCT‐based analysis, enabling motion‐informed margin design in liver SBRT.

## AUTHOR CONTRIBUTIONS

Daisuke Kawahara conceived and designed the study, and write the manuscript. Daisuke Kawahara, Hirokazu Masuda, Takuya Wada, Misato Kishi, Tsuyoshi Katsuta, and Yuji Murakami performed data collection, data analysis, and interpretation of results.

## CONFLICTS OF INTEREST STATEMENT

The authors declare no conflicts of interest.

## ETHNICAL APPROVAL

All procedures performed in studies involving human participants were in accordance with the ethical standards of the institutional and/or national research committee and with the 1964 Helsinki declaration and its later amendments or comparable ethical standards.

## INFORMED CONSENT

Informed consent was obtained from all individual participants included in the study.

## Supporting information



Supporting information
