# Supplementary material for: Margin reduction and optimal prescription isodose model for liver stereotactic radiotherapy with respiratory motion
Source: J Appl Clin Med Phys. 2026 Jan 7;27(1):e70455. doi: 10.1002/acm2.70455 (PMC12779934; doi:10.1002/acm2.70455)
Supplement: Supplementary file 1 — Supporting information [file ACM2-27-e70455-s001.docx]

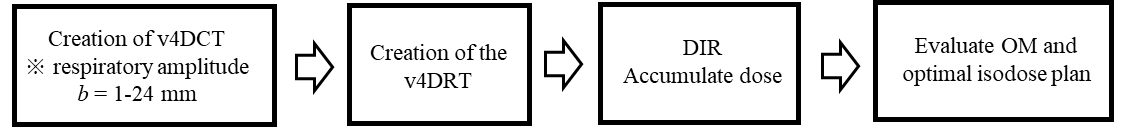


Fig. S1 Workflow for the clinical implementation of virtual four-dimensional radiotherapy (v4DRT) using v4DCT for liver SBRT. The v4DCT was generated using exhale-phase images from an existing 4DCT dataset.


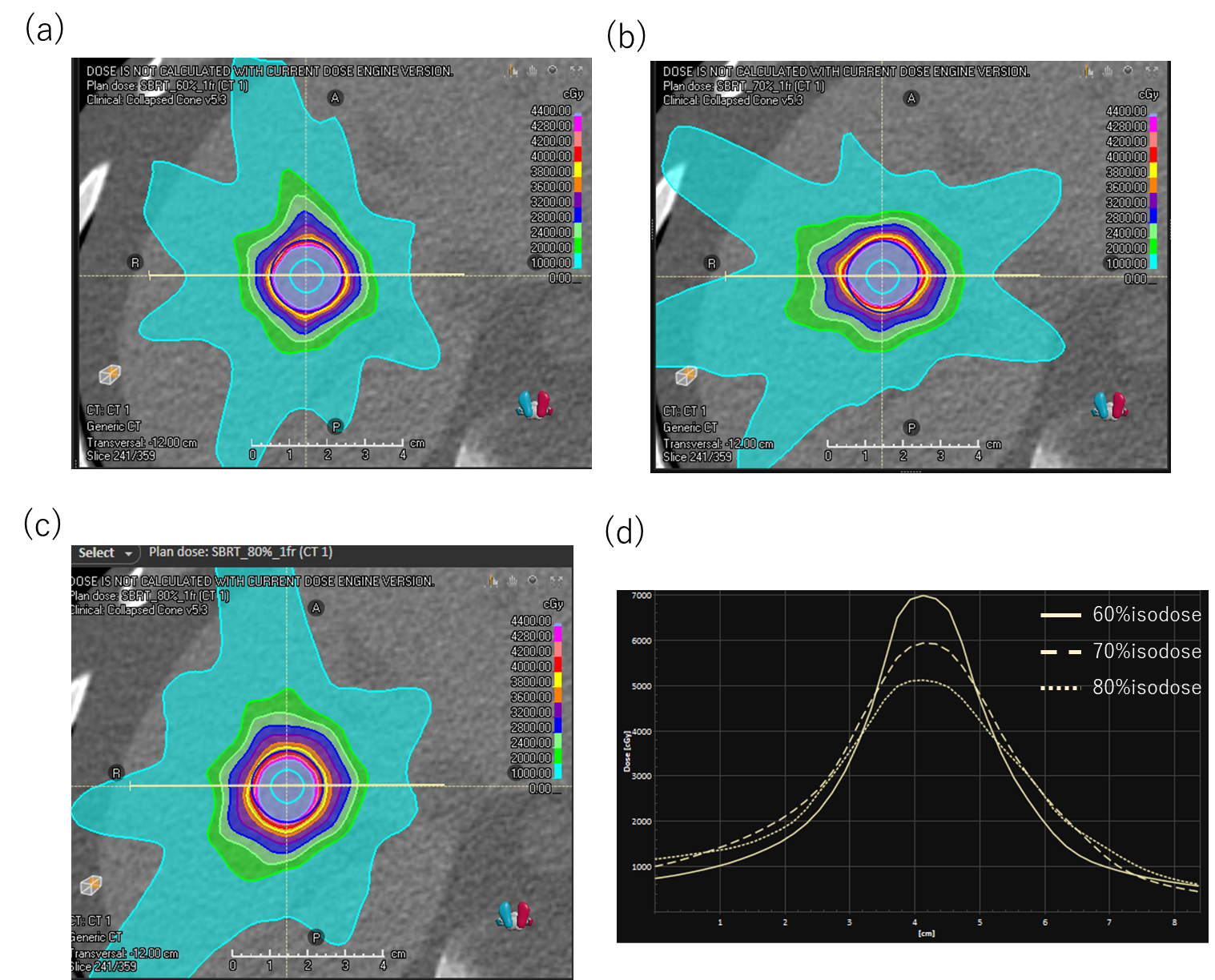


Fig. S2. Dose distributions and radial dose–falloff characteristics for the three prescription isodose levels. (a) 60% isodose prescription plan, (b) 70% isodose prescription plan, and (c) 80% isodose prescription plan shown on the same axial CT slice for a representative SBRT case. Steeper dose gradients are observed in the lower prescription plans (60% and 70%), resulting in a more compact high-dose region around the target. (d) Radial dose–falloff curves derived from the same plans, demonstrating increasingly sharper gradients for lower prescription isodose levels.


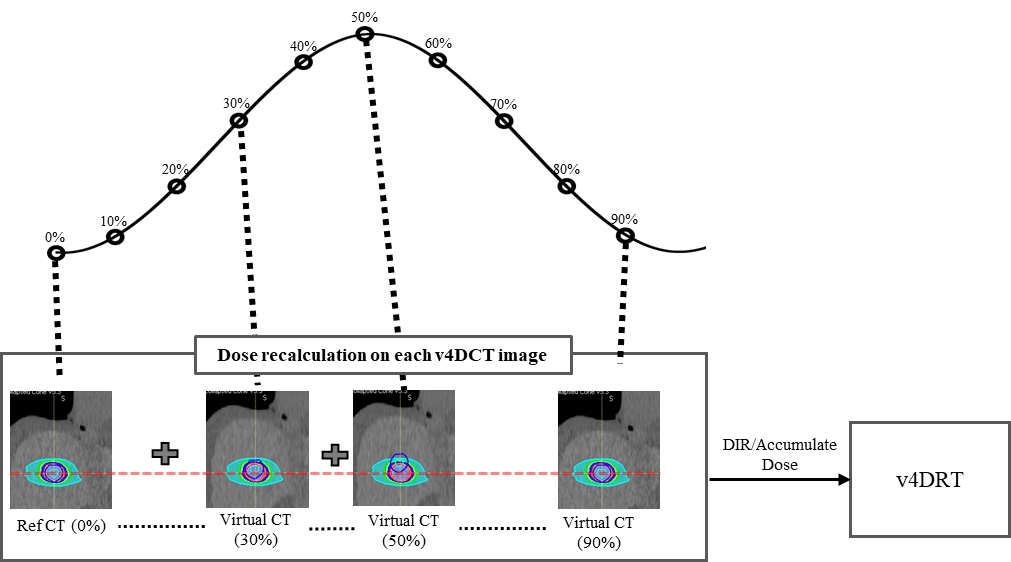


Fig. S3 Creation of the v4DCT, and v4DRT calculated with the dose distribution of the deformed v4DCT.


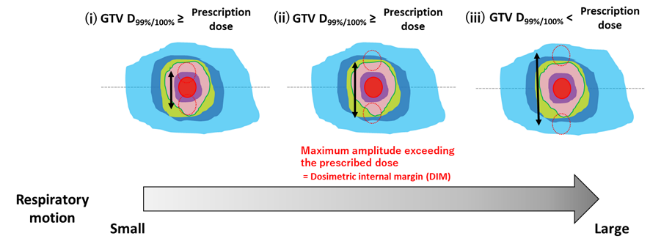


Fig. S4 Illustration of the dosimetric internal margin (DIM) and the effect of increasing respiratory motion on dose coverage. As respiratory-induced tumor motion increases from (i) to (iii), the tumor's displacement becomes more significant. When motion is within the DIM, the gross tumor volume (GTV) still receives the full prescription dose ($D_{99\%/100\%}$ ≥ prescription dose). However, when tumor motion exceeds the DIM, underdosage occurs in some tumor regions (circled in red), where $D_{99\%/100\%}$ < prescription dose, potentially compromising tumor control.


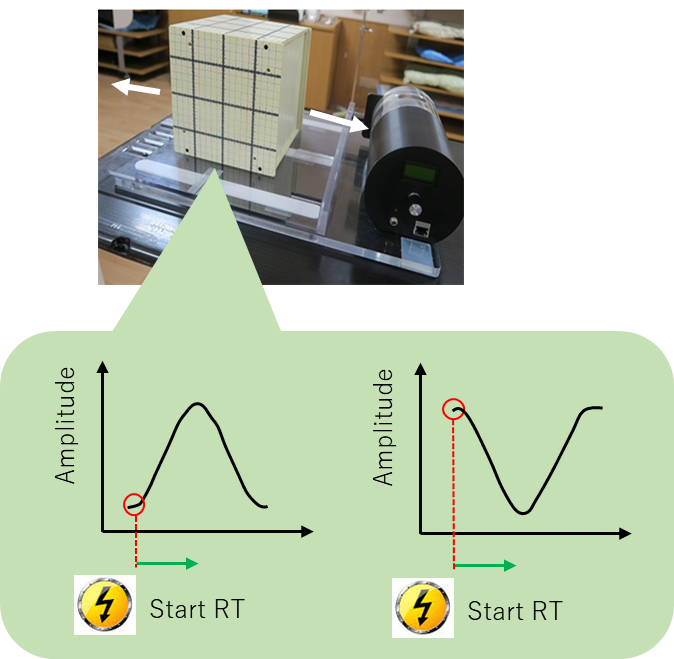


Fig. S5 Interplay effect measured by the phantom.


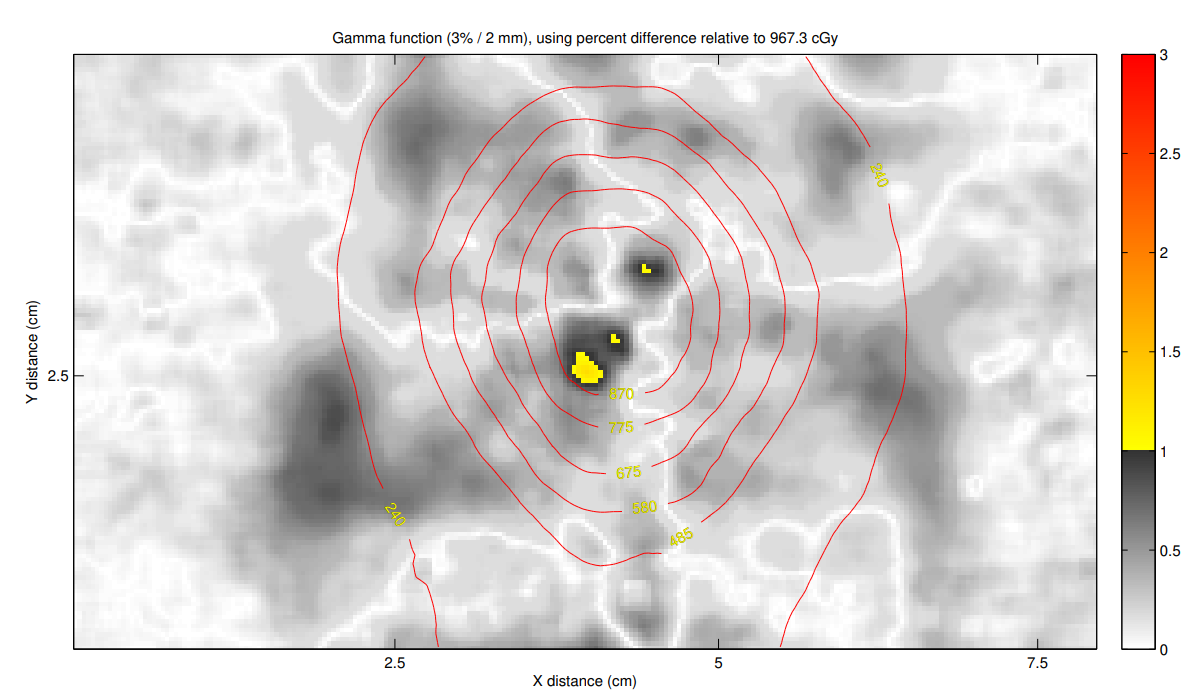


Fig. S6 Gamma analysis results for phantom irradiation under respiratory motion. The gamma maps correspond to two different initial respiratory phases (−π/2 and π/2) to assess the impact of phase-dependent interplay effects. These phases were selected to evaluate dose distribution variations caused by respiratory motion, as described in Fig. S5. Gamma analysis was performed with a 3%/3 mm global criterion to compare the planned and measured dose distributions.
